# 2,6-Dimeth­oxy-9,10-anthraquinone

**DOI:** 10.1107/S1600536812037361

**Published:** 2012-09-05

**Authors:** Akira Ohta, Kazuki Hattori, Takashi Kobayashi, Hiroyoshi Naito, Takeshi Kawase, Chitoshi Kitamura

**Affiliations:** a Department of Chemistry, Faculty of Science, Shinshu University, 3-1-1 Asahi, Matsumoto, Nagano 390-8621, Japan; b Department of Physics and Electronics, Graduate School of Engineering, Osaka Prefecture University, 1-1 Gakuencho, Naka-ku, Sakai, Osaka 599-8531, Japan; c Department of Materials Science and Chemistry, Graduate School of Engineering, University of Hyogo, 2167 Shosha, Himeji, Hyogo 671-2280, Japan

## Abstract

The title compound, C_16_H_12_O_4_, crystallizes with two half-mol­ecules in the asymmetric unit, each of which is completed by a crystallographic inversion center. The two crystallographically independent mol­ecules have almost the same geometry and are almost planar [maximum deviations = 0.018 (3) and 0.049 (3) Å]. They adopt a conformation in which the C_meth­yl_—O bonds are directed along the mol­ecular short axis [C—C—O—C torsion angles of 179.6 (2) and 178.0 (2)°]. In the crystal, the mol­ecular packing is characterized by a combination of a columnar stacking and a herringbone-like arrangement. The mol­ecules form slipped π-stacks along the *b* axis, in which there are two kinds of columns differing from each other in their slippage. The inter­planar distances between neighboring mol­ecules are 3.493 (3) for one column and 3.451 (2) Å for the other.

## Related literature

For a study of the effects of alk­oxy substituents on the structures and solid-state photophysics of anthraquinones, see: Ohta, Hattori, Kusumoto, *et al.* (2012 ▶). For the synthesis, see: Keller & Rüchardt (1998 ▶). For a related structure, see: Ohta, Hattori, Kawase, *et al.* (2012 ▶).
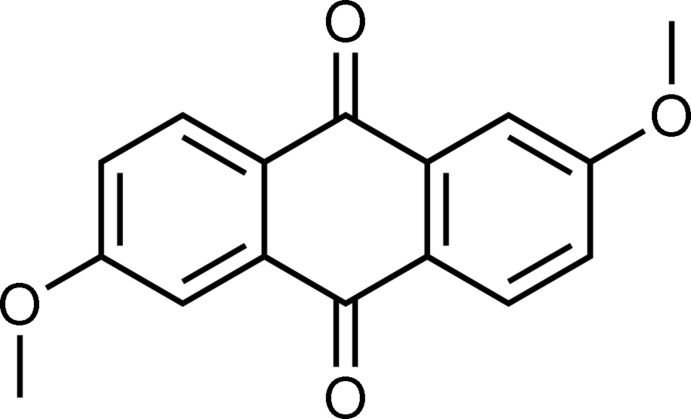


## Experimental

### 

#### Crystal data

C_16_H_12_O_4_
*M* *_r_* = 268.26
Monoclinic, 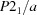

*a* = 16.2689 (19) Å
*b* = 3.9357 (4) Å
*c* = 19.9510 (19) Å
β = 109.499 (3)°
*V* = 1204.2 (2) Å^3^
*Z* = 4
Mo *K*α radiation
μ = 0.11 mm^−1^
*T* = 223 K
0.58 × 0.08 × 0.06 mm

#### Data collection

Rigaku R-AXIS RAPID diffractometer
10392 measured reflections
2743 independent reflections
1501 reflections with *I* > 2σ(*I*)
*R*_int_ = 0.066

#### Refinement

*R*[*F* ^2^ > 2σ(*F* ^2^)] = 0.057
*wR*(*F* ^2^) = 0.178
*S* = 1.00
2743 reflections
183 parameters
H-atom parameters constrained
Δρ_max_ = 0.26 e Å^−3^
Δρ_min_ = −0.28 e Å^−3^

### 

Data collection: *RAPID-AUTO* (Rigaku, 1999 ▶); cell refinement: *PROCESS-AUTO* (Rigaku, 1998 ▶); data reduction: *PROCESS-AUTO*; program(s) used to solve structure: *SIR2004* (Burla *et al.*, 2005 ▶); program(s) used to refine structure: *SHELXL97* (Sheldrick, 2008 ▶); molecular graphics: *ORTEP-3 for Windows* (Farrugia, 1997 ▶); software used to prepare material for publication: *WinGX* (Farrugia, 1999 ▶).

## Supplementary Material

Crystal structure: contains datablock(s) global, I. DOI: 10.1107/S1600536812037361/kj2210sup1.cif

Structure factors: contains datablock(s) I. DOI: 10.1107/S1600536812037361/kj2210Isup2.hkl

Supplementary material file. DOI: 10.1107/S1600536812037361/kj2210Isup3.cml

Additional supplementary materials:  crystallographic information; 3D view; checkCIF report

## Figures and Tables

**Fig. 1.**
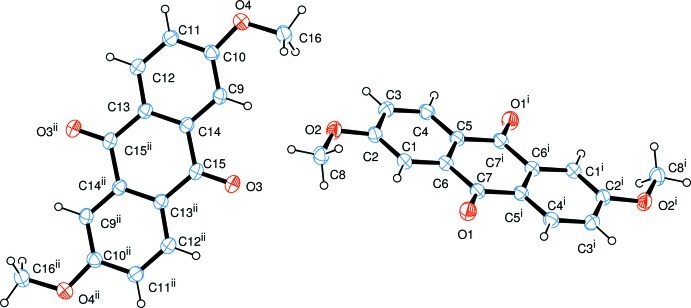
The molecular structure of the title compound, showing the atomic numbering and 50% probability displacement ellipsoids. Symmetry code: (i) -x, -y + 1, -z. (ii) -x + 1, -y + 1, -z + 1.

**Fig. 2.**
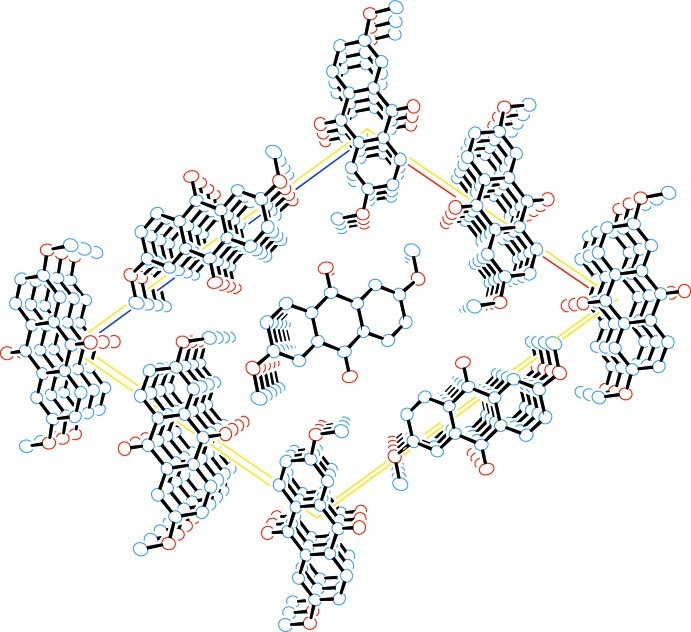
The packing diagram of the title compound, viewed down the b axis. Hydrogen atoms are omitted for clarity.

## supplementary crystallographic information

### Comment

9,10-Anthraquinone is an important building block of many dyes and pigments. Recently, we have investigated the effects of alkoxy substituents on the optical properties of anthraquinones both in solution and in the solid state (Ohta, Hattori, Kusumoto, *et al.*, 2012). We have revealed the cystal structure of 2,3,6,7-tetramethoxy-9,10- anthraquinone (Ohta, Hattori, Kawase, *et al.*, 2012). In order to elucidate the substitution effects of the methoxy groups on the crystal packing, the X-ray analysis of the title compound was performed.

The molecular structure of the title compound is shown in Fig. 1. The title compound crystallizes with two halves of the molecule in the asymmetric unit of the unit cell. The complete molecules are located on crystallographic inversion centers. The molecules are almost planar with the maximum deviation of 0.018 (3) Å for C8 in one molecule and 0.049 (3) Å for C16 in another molecule. The molecules prefer the conformations in which the C_methyl_—O bonds are directed along the short molecular axis. Thus, the torsion angles of C3—C2—O2—C8 and C11—C10—O4—C16 are 179.6 (2) and 178.0 (2)°, respectively. Theses conformations are similar to the coressponding moiety in 2,3,6,7-tetramethoxy-9,10-anthraquinone (Ohta, Hattori, Kawase, *et al.*, 2012). However, there is a large difference in crystal packing between the title compound and 2,3,6,7-tetramethoxy-9,10-anthraquinone. As shown in Fig. 2, the crystal structure is characterized by a columnar stacking and a herrinbone-like arrangement, although 2,3,6,7-tetramethoxy- 9,10-anthraquinone molecules took a slipped-parallel arrangement. Along the *b* axis, there are two columns in which molecules form slipped π-stacks. The interplanar distances between neighboring molecules are 3.493 (3) Å for one column and 3.451 (2) Å for another column. Furthermore, the translational shifts of neighboring molecules in the stacks are as follows: For molecule 1 (C1–C8, O1, and O2), the slip distance between neighboring molecules is 3.94 Å, and the anthraquinone rings in the column slipped relative to each other along the long molecular axis by 0.59 Å and along the short molecular axis by 1.72 Å. In contrast, for molecules 2 (C9–C16, O3, and O4), the slip distance between neighboring molecules is 3.94 Å, and the anthraquinone rings in the column slipped relative to each other along the long molecular axis by 1.89 Å and along the short molecular axis by 0.05 Å.

To examine the influence of crystal packing on the solid-state fluorescence, the fluorescence spectrum and the absolute quantum yield of the title compound were measured with a Hamamatsu Photonics PMA11 calibrated optical multichannel analyzer with a solid-state blue laser (*λ*_ex_ = 377 nm) and a Labsphere IS IS-040-SF integrating sphere, respectively. The crystals showed negligible fluorescence (*Φ* < 0.001). The fluorescence quenching may result from the π-stacked structure.

### Experimental

The title compound was prepared according to the literature procedure (Keller & Rüchardt, 1998). Single crystals suitable for X-ray analysis were obtained by recrystallization from toluene.

### Refinement

All the H atoms were positioned geometrically and refined using a riding model with C—H = 0.94 Å and *U*_iso_(H) = 1.2*U*_eq_(C) for aromatic C—H, and C—H = 0.97 Å and *U*_iso_(H) = 1.5*U*_eq_(C) for CH_3_. The positions of methyl H atoms were optimized rotationally.

### Crystal data

**Table:**

C_16_H_12_O_4_	*F*(000) = 560
*M**_r_* = 268.26	*D*_x_ = 1.48 Mg m^−^^3^
Monoclinic, *P*2_1_/*a*	Mo *K*α radiation, λ = 0.71073 Å
Hall symbol: -P 2yab	Cell parameters from 5015 reflections
*a* = 16.2689 (19) Å	θ = 3.2–27.5°
*b* = 3.9357 (4) Å	µ = 0.11 mm^−^^1^
*c* = 19.9510 (19) Å	*T* = 223 K
β = 109.499 (3)°	Prism, yellow
*V* = 1204.2 (2) Å^3^	0.58 × 0.08 × 0.06 mm
*Z* = 4	

### Data collection

**Table:**

Rigaku R-AXIS RAPID diffractometer	1501 reflections with *I* > 2σ(*I*)
Radiation source: fine-focus sealed x-ray tube	*R*_int_ = 0.066
Graphite monochromator	θ_max_ = 27.5°, θ_min_ = 3.2°
Detector resolution: 10 pixels mm^-1^	*h* = −21→21
ω scans	*k* = −4→5
10392 measured reflections	*l* = −22→25
2743 independent reflections	

### Refinement

**Table:**

Refinement on *F*^2^	Primary atom site location: structure-invariant direct methods
Least-squares matrix: full	Secondary atom site location: difference Fourier map
*R*[*F*^2^ > 2σ(*F*^2^)] = 0.057	Hydrogen site location: inferred from neighbouring sites
*wR*(*F*^2^) = 0.178	H-atom parameters constrained
*S* = 1.00	*w* = 1/[σ^2^(*F*_o_^2^) + (0.0915*P*)^2^] where *P* = (*F*_o_^2^ + 2*F*_c_^2^)/3
2743 reflections	(Δ/σ)_max_ < 0.001
183 parameters	Δρ_max_ = 0.26 e Å^−^^3^
0 restraints	Δρ_min_ = −0.28 e Å^−^^3^

### Special details

**Table:**

Geometry. All s.u.'s (except the s.u. in the dihedral angle between two l.s. planes) are estimated using the full covariance matrix. The cell s.u.'s are taken into account individually in the estimation of s.u.'s in distances, angles and torsion angles; correlations between s.u.'s in cell parameters are only used when they are defined by crystal symmetry. An approximate (isotropic) treatment of cell s.u.'s is used for estimating s.u.'s involving l.s. planes.
Refinement. Refinement of *F*^2^ against ALL reflections. The weighted *R*-factor *wR* and goodness of fit *S* are based on *F*^2^, conventional *R*-factors *R* are based on *F*, with *F* set to zero for negative *F*^2^. The threshold expression of *F*^2^ > 2σ(*F*^2^) is used only for calculating *R*-factors(gt) *etc*. and is not relevant to the choice of reflections for refinement. *R*-factors based on *F*^2^ are statistically about twice as large as those based on *F*, and *R*- factors based on ALL data will be even larger.

### Fractional atomic coordinates and isotropic or equivalent isotropic displacement parameters (Å^2^)

**Table:**

	*x*	*y*	*z*	*U*_iso_*/*U*_eq_	
C1	0.10535 (16)	0.2528 (6)	0.13534 (12)	0.0412 (6)	
H1	0.0785	0.1497	0.1652	0.049*	
C2	0.19519 (17)	0.2826 (6)	0.15742 (12)	0.0424 (6)	
C3	0.23494 (17)	0.4388 (6)	0.11278 (12)	0.0452 (6)	
H3	0.296	0.4582	0.1275	0.054*	
C4	0.18464 (17)	0.5631 (6)	0.04772 (12)	0.0444 (6)	
H4	0.2117	0.6696	0.0184	0.053*	
C5	0.09401 (16)	0.5337 (6)	0.02449 (11)	0.0387 (6)	
C6	0.05487 (16)	0.3753 (6)	0.06904 (11)	0.0389 (6)	
C7	−0.04125 (16)	0.3291 (6)	0.04555 (11)	0.0416 (6)	
C8	0.21230 (19)	0.0119 (7)	0.26849 (13)	0.0522 (7)	
H8A	0.179	−0.1854	0.2455	0.078*	
H8B	0.1741	0.1718	0.2805	0.078*	
H8C	0.2581	−0.0583	0.3115	0.078*	
O1	−0.07545 (12)	0.1767 (5)	0.08334 (8)	0.0541 (5)	
O2	0.25054 (12)	0.1718 (5)	0.22088 (8)	0.0506 (5)	
C9	0.47309 (16)	0.1926 (6)	0.36503 (12)	0.0401 (6)	
H9	0.4168	0.1898	0.331	0.048*	
C10	0.54271 (17)	0.0488 (6)	0.34963 (12)	0.0424 (6)	
C11	0.62617 (17)	0.0536 (6)	0.39982 (12)	0.0444 (6)	
H11	0.6731	−0.0422	0.3888	0.053*	
C12	0.64007 (17)	0.1983 (6)	0.46545 (12)	0.0430 (6)	
H12	0.6965	0.1982	0.4993	0.052*	
C13	0.57121 (15)	0.3458 (6)	0.48250 (11)	0.0380 (6)	
C14	0.48757 (15)	0.3411 (6)	0.43153 (11)	0.0377 (5)	
C15	0.41234 (15)	0.4966 (6)	0.44723 (12)	0.0409 (6)	
C16	0.45117 (19)	−0.1244 (8)	0.23357 (13)	0.0556 (7)	
H16A	0.4122	−0.2454	0.2529	0.083*	
H16B	0.4552	−0.2453	0.1924	0.083*	
H16C	0.4287	0.1026	0.2196	0.083*	
O3	0.33925 (12)	0.4938 (5)	0.40245 (8)	0.0542 (5)	
O4	0.53622 (12)	−0.1021 (5)	0.28663 (8)	0.0520 (5)	

### Atomic displacement parameters (Å^2^)

**Table:**

	*U*^11^	*U*^22^	*U*^33^	*U*^12^	*U*^13^	*U*^23^
C1	0.0444 (15)	0.0426 (13)	0.0369 (11)	−0.0015 (11)	0.0141 (10)	−0.0030 (10)
C2	0.0446 (15)	0.0446 (13)	0.0363 (11)	0.0018 (11)	0.0110 (10)	−0.0005 (10)
C3	0.0375 (14)	0.0536 (15)	0.0446 (13)	0.0014 (11)	0.0137 (11)	0.0008 (12)
C4	0.0445 (15)	0.0486 (14)	0.0421 (13)	−0.0036 (11)	0.0172 (11)	−0.0037 (11)
C5	0.0400 (14)	0.0402 (12)	0.0369 (11)	−0.0008 (10)	0.0144 (10)	−0.0015 (10)
C6	0.0425 (14)	0.0399 (12)	0.0361 (11)	−0.0016 (10)	0.0154 (10)	−0.0019 (10)
C7	0.0439 (15)	0.0441 (14)	0.0374 (12)	−0.0019 (11)	0.0145 (11)	−0.0030 (11)
C8	0.0523 (17)	0.0594 (16)	0.0422 (13)	0.0005 (13)	0.0122 (12)	0.0069 (12)
O1	0.0466 (11)	0.0721 (12)	0.0454 (9)	−0.0061 (9)	0.0177 (8)	0.0096 (9)
O2	0.0437 (11)	0.0645 (12)	0.0410 (9)	0.0022 (8)	0.0106 (8)	0.0088 (8)
C9	0.0391 (14)	0.0429 (13)	0.0351 (11)	0.0013 (10)	0.0081 (9)	0.0056 (10)
C10	0.0459 (15)	0.0433 (13)	0.0386 (12)	0.0012 (11)	0.0149 (11)	0.0013 (11)
C11	0.0416 (15)	0.0480 (14)	0.0446 (13)	0.0034 (11)	0.0158 (11)	0.0052 (11)
C12	0.0367 (14)	0.0455 (13)	0.0448 (13)	0.0014 (10)	0.0109 (10)	0.0042 (11)
C13	0.0346 (13)	0.0396 (12)	0.0388 (12)	0.0009 (10)	0.0110 (10)	0.0053 (10)
C14	0.0354 (13)	0.0376 (12)	0.0389 (11)	0.0014 (10)	0.0108 (10)	0.0075 (10)
C15	0.0354 (14)	0.0425 (13)	0.0405 (12)	0.0011 (10)	0.0068 (10)	0.0066 (11)
C16	0.0592 (19)	0.0597 (17)	0.0421 (13)	0.0018 (13)	0.0094 (12)	−0.0066 (13)
O3	0.0367 (11)	0.0701 (12)	0.0484 (10)	0.0058 (9)	0.0042 (8)	−0.0043 (9)
O4	0.0524 (12)	0.0620 (11)	0.0413 (9)	0.0031 (9)	0.0153 (8)	−0.0049 (8)

### Geometric parameters (Å, º)

**Table:**

C1—C2	1.384 (4)	C9—C10	1.389 (3)
C1—C6	1.390 (3)	C9—C14	1.396 (3)
C1—H1	0.94	C9—H9	0.94
C2—O2	1.357 (3)	C10—O4	1.362 (3)
C2—C3	1.405 (3)	C10—C11	1.392 (3)
C3—C4	1.373 (3)	C11—C12	1.376 (3)
C3—H3	0.94	C11—H11	0.94
C4—C5	1.395 (4)	C12—C13	1.401 (3)
C4—H4	0.94	C12—H12	0.94
C5—C6	1.401 (3)	C13—C14	1.401 (3)
C5—C7^i^	1.477 (3)	C13—C15^ii^	1.474 (3)
C6—C7	1.486 (3)	C14—C15	1.492 (3)
C7—O1	1.232 (3)	C15—O3	1.226 (3)
C7—C5^i^	1.477 (3)	C15—C13^ii^	1.474 (3)
C8—O2	1.442 (3)	C16—O4	1.437 (3)
C8—H8A	0.97	C16—H16A	0.97
C8—H8B	0.97	C16—H16B	0.97
C8—H8C	0.97	C16—H16C	0.97
			
C2—C1—C6	120.0 (2)	C10—C9—C14	119.3 (2)
C2—C1—H1	120	C10—C9—H9	120.3
C6—C1—H1	120	C14—C9—H9	120.3
O2—C2—C1	124.8 (2)	O4—C10—C9	124.3 (2)
O2—C2—C3	115.4 (2)	O4—C10—C11	115.2 (2)
C1—C2—C3	119.7 (2)	C9—C10—C11	120.4 (2)
C4—C3—C2	120.0 (2)	C12—C11—C10	120.1 (2)
C4—C3—H3	120	C12—C11—H11	120
C2—C3—H3	120	C10—C11—H11	120
C3—C4—C5	121.0 (2)	C11—C12—C13	120.8 (2)
C3—C4—H4	119.5	C11—C12—H12	119.6
C5—C4—H4	119.5	C13—C12—H12	119.6
C4—C5—C6	118.7 (2)	C12—C13—C14	118.7 (2)
C4—C5—C7^i^	120.0 (2)	C12—C13—C15^ii^	120.0 (2)
C6—C5—C7^i^	121.3 (2)	C14—C13—C15^ii^	121.3 (2)
C1—C6—C5	120.6 (2)	C9—C14—C13	120.7 (2)
C1—C6—C7	118.9 (2)	C9—C14—C15	118.8 (2)
C5—C6—C7	120.5 (2)	C13—C14—C15	120.6 (2)
O1—C7—C5^i^	121.2 (2)	O3—C15—C13^ii^	121.4 (2)
O1—C7—C6	120.6 (2)	O3—C15—C14	120.5 (2)
C5^i^—C7—C6	118.2 (2)	C13^ii^—C15—C14	118.1 (2)
O2—C8—H8A	109.5	O4—C16—H16A	109.5
O2—C8—H8B	109.5	O4—C16—H16B	109.5
H8A—C8—H8B	109.5	H16A—C16—H16B	109.5
O2—C8—H8C	109.5	O4—C16—H16C	109.5
H8A—C8—H8C	109.5	H16A—C16—H16C	109.5
H8B—C8—H8C	109.5	H16B—C16—H16C	109.5
C2—O2—C8	117.2 (2)	C10—O4—C16	117.7 (2)
			
C6—C1—C2—O2	−179.9 (2)	C14—C9—C10—O4	−179.9 (2)
C6—C1—C2—C3	0.4 (4)	C14—C9—C10—C11	−0.2 (4)
O2—C2—C3—C4	−179.3 (2)	O4—C10—C11—C12	−179.7 (2)
C1—C2—C3—C4	0.4 (4)	C9—C10—C11—C12	0.6 (4)
C2—C3—C4—C5	−0.7 (4)	C10—C11—C12—C13	−0.7 (4)
C3—C4—C5—C6	0.2 (3)	C11—C12—C13—C14	0.4 (4)
C3—C4—C5—C7^i^	179.7 (2)	C11—C12—C13—C15^ii^	−179.2 (2)
C2—C1—C6—C5	−0.9 (4)	C10—C9—C14—C13	−0.1 (3)
C2—C1—C6—C7	177.9 (2)	C10—C9—C14—C15	179.6 (2)
C4—C5—C6—C1	0.6 (3)	C12—C13—C14—C9	0.0 (3)
C7^i^—C5—C6—C1	−178.9 (2)	C15^ii^—C13—C14—C9	179.6 (2)
C4—C5—C6—C7	−178.1 (2)	C12—C13—C14—C15	−179.7 (2)
C7^i^—C5—C6—C7	2.4 (4)	C15^ii^—C13—C14—C15	−0.1 (4)
C1—C6—C7—O1	−1.9 (3)	C9—C14—C15—O3	−0.1 (3)
C5—C6—C7—O1	176.8 (2)	C13—C14—C15—O3	179.6 (2)
C1—C6—C7—C5^i^	178.9 (2)	C9—C14—C15—C13^ii^	−179.6 (2)
C5—C6—C7—C5^i^	−2.3 (4)	C13—C14—C15—C13^ii^	0.1 (4)
C1—C2—O2—C8	−0.1 (3)	C9—C10—O4—C16	−2.3 (3)
C3—C2—O2—C8	179.6 (2)	C11—C10—O4—C16	178.0 (2)

Symmetry codes: (i) −*x*, −*y*+1, −*z*; (ii) −*x*+1, −*y*+1, −*z*+1.
